# High altitude-related hypertensive crisis and acute kidney injury in an asymptomatic healthy individual

**DOI:** 10.1186/s13728-016-0051-3

**Published:** 2016-09-14

**Authors:** Edward Gilbert-Kawai, Daniel Martin, Michael Grocott, Denny Levett

**Affiliations:** 1 Centre for Altitude Space and Extreme Environment Medicine, University College London, UCLH NIHR Biomedical Research Centre, Institute of Sport and Exercise Health, 170 Tottenham Court Road, London, W1T 7HA UK; 2 Integrative Physiology and Critical Illness Group, Clinical and Experimental Sciences, University of Southampton, Southampton, SO16 6YD UK

**Keywords:** High altitude, Hypertension, Acute kidney injury

## Abstract

**Background:**

High-altitude exposure causes a mild to moderate rise in systolic and diastolic blood pressure. This case report describes the first documented case of a hypertensive crisis at altitude, as well as the first report of the occurrence of acute kidney injury in the context of altitude-related hypertension.

**Case presentation:**

A healthy, previously normotensive 30-year old, embarked on a trek to Everest Base Camp (5300 m). During his 11-day ascent the subject developed increasingly worsening hypertension. In the absence of symptoms, the individual initially elected to remain at altitude as had previously been the plan. However, an increase in the severity of his hypertension to a peak of 223/119 mmHg resulted in a decision to descend. On descent he was found to have an acute kidney injury that subsequently resolved spontaneously. His blood pressure reverted to normal at sea level and subsequent investigations including a transthoracic echocardiogram, cardiac magnetic resonance imaging, renal ultrasound, and urinary catecholamines were normal.

**Conclusion:**

This report challenges the view that transient rises in blood pressure at altitude are without immediate risk. We review the evidence that altitude induces hypertension and discuss the implications for the management of hypertension at altitude.

## Background

Hypertension is defined as a blood pressure (BP) above 140/90 mmHg [1]. Acute exposure to high altitude causes a mild-to-moderate increase in both systolic (SBP) and diastolic (DBP) blood pressure although there is marked inter-individual variability in the response [2, 3]. The increase is usually transient, and is evident in both healthy individuals and those with pre-existing hypertension [4]. ‘Severe hypertension’ is defined as having a systolic BP ≥ 180 mmHg, or a diastolic BP ≥ 110 mmHg. Acute severe hypertension is also referred to as a ‘hypertensive crises’ or ‘hypertensive emergency’ if associated with acute end-organ damage, or as ‘hypertensive urgency’ in the absence of acute end-organ damage [1, 5]. This case report describes the development of a hypertensive crisis with acute kidney injury in a previously healthy 30-year old. Implications for the management of hypertension at high altitude are discussed.

## Case presentation

A 30-year-old Caucasian male, normally resident at sea level with no previous medical history, embarked on a trek to Everest Base Camp (EBC) (5300 m) as an investigator on the Xtreme Everest 2 research expedition [6]. His blood pressure had been measured at sea level as part of the physiological research investigations and he was normotensive (126/82 mmHg). He had no cardiovascular risk factors for hypertension. As part of the data collection during the research expedition his blood pressure, heart rate, respiratory rate and the saturation of peripheral arterial blood with oxygen (‘oxygen saturation’) were measured in a standardized fashion each morning after 5 min of seated rest [7] (Table 1). The subject was blinded to the blood pressure measurements, whereby the mean of three resting readings was recorded (M3W, Omron, Japan), and oxygen saturation was measured using a pulse oximeter placed on the individual’s dominant forefinger (Nonin Onyx, Minnesota, USA).

**Table 1:** Physiological variables at rest during ascent to altitude

Day (altitude in metres)	Blood pressure (mmHg)	Heart rate (beats/min)	Ventilatory rate (breaths/min)	SpO_2_ (%)
0 (50)	126/82	64	9	99
1 (1300)	132/87	68	13	98
2 (2850)	134/88	66	15	96
3 (3500)	146/91	68	10	93
4 (3500)	158/93	70	13	94
5 (3500)	151/91	64	8	92
6 (3770)	140/93	83	9	89
7 (4250)	147/95	68	13	90
8 (4250)	142/84	72	11	84
9 (4250)	157/93	64	11	86
10 (4950)	148/96	76	10	88
11 (5160)	143/92	71	11	82
12 (5300)	146/97	71	14	76
13 (5300)	167/106	63	15	77

Subjects initially flew from London (day 0) to Kathmandu (day 1). They then flew to Lukla (2850 m) (day 2) before trekking to Everest Base Camp (5300 m) over 11 days

During his ascent to EBC, the subject reported minimal symptoms of acute mountain sickness (AMS) that did not require treatment, and was otherwise well. The subject was reviewed by the expedition medical officer on arrival at EBC because his hypertension was persistent. To confirm the accuracy of the blood pressure readings taken with the automated oscillometric blood pressure monitor, measurements were made with another electronic device (Tango+, SunTech Medical, USA) and using a manual sphygmomanometer. Since the three methods produced consistent results (observed to be within 5 mmHg of each other on repeated recordings), the Omron was subsequently used for all measurements. The subject remained asymptomatic and physical examination, electrocardiogram (ECG) and urine analysis were normal. Unfortunately due to equipment failure we were unable to examine the subject for signs of retinal haemorrhage or papilloedema. In the absence of symptoms, and after discussion with the expedition medical team and a review of the literature, he initially decided to remain at altitude with twice-daily blood pressure monitoring in the expectation that the increase in BP would be transient as has previously been reported [8].

**Fig. 1:**
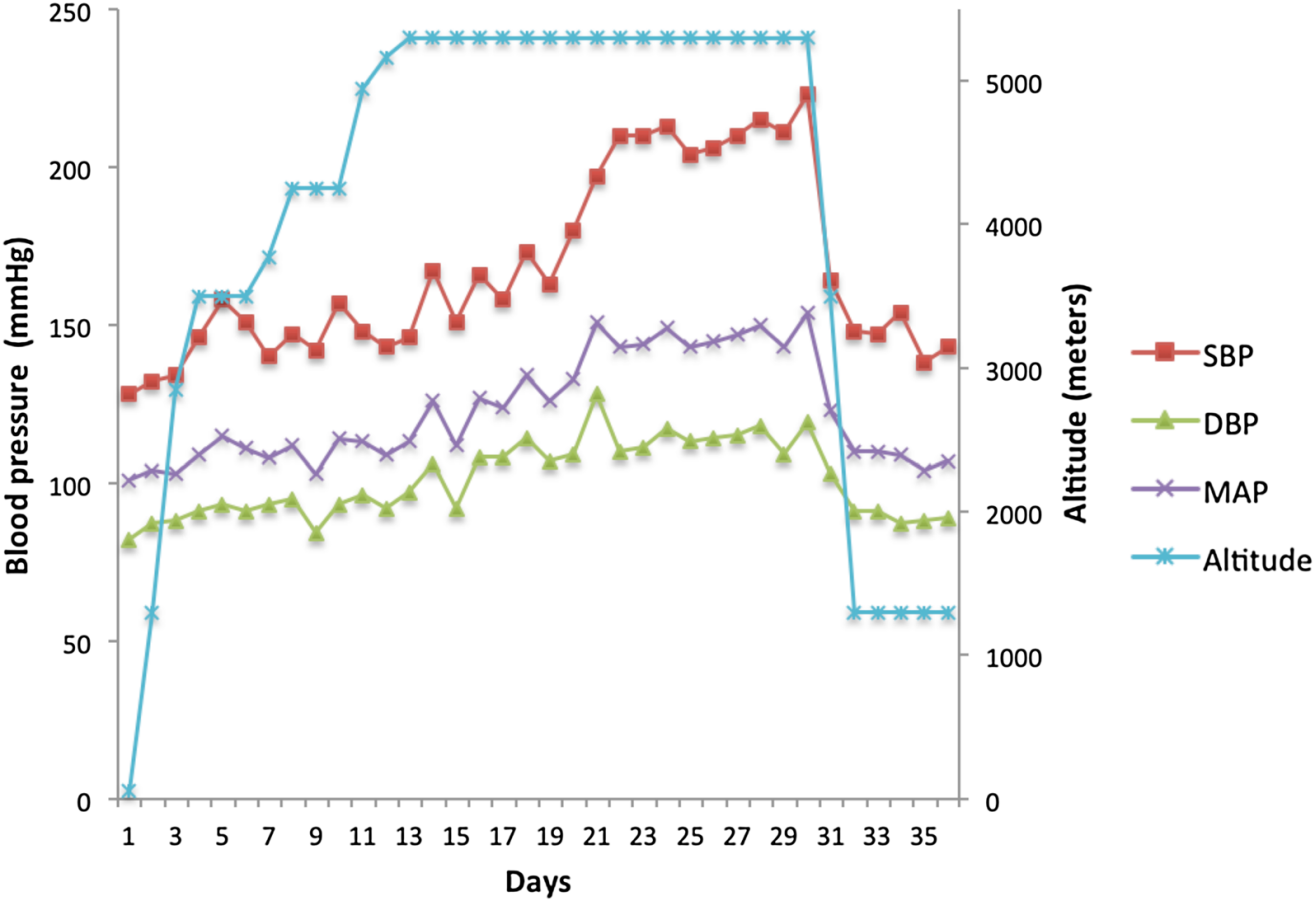
Subject’s systolic, diastolic and mean arterial blood pressure throughout the expedition. *SBP* systolic blood pressure, *DBP* diastolic blood pressure, *MAP* mean arterial pressure

After 5 days at EBC, the subject’s diastolic BP increased above 110 mmHg (173/114), and a diagnosis of severe hypertension was made (see Fig. 1). Whilst his DBP decreased on the subsequent 2 days, (107 and 109 mmHg), his SBP continued to increase reaching 180 mmHg by day seven at EBC. A peak blood pressure of 223/119 mmHg was recorded on day seventeen at EBC. Although he remained asymptomatic, end-organ damage could not be excluded in the field. As a result, the decision was made that he should descend back to Kathmandu for further investigation and for the instigation of treatment as required.

The subject’s blood pressure improved on descent to Kathmandu, but he remained hypertensive initially with a resting blood pressure of 148/91 mmHg. Physical examination was normal as was his ECG. Blood results showed evidence of acute kidney injury (AKI) with a urea of 16.2 mmol/L and a creatinine of 168 μmol/L [9], and urine dipstix demonstrated ‘2+’ of protein (corresponding to a semi-quantitative value of 100 g/l), and ‘2+’ of blood. Using the Cockcroft–Gault equation, his estimated creatinine clearance was 68.3 ml/min [10]. Further investigations included an echocardiogram (ECHO) ‘trace mitral regurgitation, trace tricuspid regurgitation, otherwise normal’, and renal ultrasound ‘normal renal Doppler study with no evidence to suggest renal artery stenosis’. Repeat testing 1 week after descent demonstrated a blood pressure of 143/89 mmHg, and normal renal function (creatinine 80 μmol/L, urea 6.8 mmol/L). Investigations for other secondary causes of hypertension were not available in Nepal and with his return to London imminent further follow-up was delayed until his repatriation. Investigations in the UK revealed normal 24-h BP monitoring (mean BP 124/84 mmHg), normal transthoracic ECHO and cardiac magnetic resonance imaging (MRI), a normal renal ultrasound, normal renal function, normal urinary catecholamines and no abnormalities on fundoscopy.

## Discussion

This is the first report of a hypertensive crisis at altitude and the first report of AKI occurring in the context of hypertension at altitude. The case illustrates an individual who experienced large and persistent increases in both SBP and DBP on exposure to high altitude with subsequent reversion of the values towards normal upon descent to lower altitude. Furthermore, this cautionary case suggests that contrary to current opinion [2], altitude-induced hypertension may be associated with end-organ damage and it is plausible that this relationship may be causal. It is noteworthy that our subject was asymptomatic so had he not been a research subject his renal impairment may have progressed unchecked. Renal pathology per se may be a cause of malignant hypertension, and renal parenchymal disease accounts for up to 80 % of all secondary causes at sea level [11]. Consequently, it is possible that the renal dysfunction occurred first causing a secondary increase in blood pressure. We consider this scenario unlikely as the subject had normal renal function prior to departure (creatinine 88 μmol/L, urea 6.8 mmol/L), and his renal function returned to normal 1 week after returning to Kathmandu when his blood pressure normalized. Moreover, acute renal failure secondary to increased hydrostatic pressures is a common sequelae of hypertensive crises [12].

Whilst the prevalence of hypertension in North American and European adults has been reported as high as 44 % [13, 14], comparatively little information exists describing the effects of altitude on blood pressure. Furthermore, the existing literature is inconsistent. In non-hypertensive patients acutely exposed to hypoxia, several studies demonstrate increased pressures [15–17], and others describe decreased pressures [8], whilst some report no change [18, 19]. Similarly in patients with pre-existing hypertension, numerous studies report elevations in blood pressure upon acute exposure to varying altitudes [17, 20–23] yet others’ reports no significant change [17, 18, 24]. In 2009, the literature was summarized [2], and having taken into account the discrepancies between study methodologies, the author concluded that whilst it was difficult to draw conclusions about blood pressure responses in hypertensive patients travelling to elevations above 3500 m, acute ascent to high altitude below this height causes a modest rise in blood pressure in patients with pre-existing mild to moderate hypertensions.

The risks of altitude-induced hypertension are currently unclear. There is no evidence to support an association between increased pressures and hypertensive complications such as hypertensive retinopathy, intracranial bleeding, or myocardial infarction [2, 22–24] or AMS [2]. However, the available data are very limited and the absence of published data should not be interpreted as implying that there is no increased risk. Consequently, the risk of short- and long-term complications of hypertensive episodes at altitude cannot, currently, be reliably estimated.

Inconsistencies in the current literature may reflect marked inter-individual variability in the hypertensive response to altitude—a point highlighted recently in the Consensus statement of the Medical Commission of the Union Internationale des Associations d’Alpinisme [25]. Furthermore, it is difficult to separate the effect of altitude on blood pressure from the multitude of other potentially confounding factors including ascent rate, altitude sickness, dehydration, temperature and stress associated with an environmental change [4, 25, 26]. Consequently, a large prospective dataset of both normotensive and hypertensive individuals with a controlled ascent profile (hypoxic exposure) is required to clarify the altitude threshold, incidence, severity, duration and reversibility of any hypertensive effect. A study of this nature would also permit further evaluation of both the cardiovascular risk and the risk of end-organ damage with altitude-related hypertension [11]. Such evidence is not currently available, but in the context of expanding tourism with an increasingly elderly tourist population, the need for accurate data to direct treatment for known hypertensives and to permit valid risk counselling prior to travel is increasingly important [2].

At sea level, guidelines for the management of severe hypertension suggest starting antihypertensive drug treatment immediately, and same day, after hospital admission if signs of papilloedema and/or retinal haemorrhage (hypertensive retinopathy) are present [1]. At altitude the prevalence of severe hypertension is unknown and there are no guidelines to direct treatment. There is evidence to support the occurrence of hypertension at altitude, but the literature is inconsistent and the precise incidence and severity of the hypertensive response to altitude are not known [3, 27, 28]. Furthermore, the implications of altitude-induced hypertension for cardiovascular risk and end-organ damage require clarification.

Unfortunately, we are unable to accurately predict which individuals will respond in this way to altitude and screening all individuals at altitude would be impractical. However, monitoring blood pressure and possibly medication adjustments should be recommended in some individuals. Certainly any individual with poorly controlled or labile hypertension or who has previously had a marked hypertensive response to altitude should be encouraged to self-monitor during ascent to altitude. Such patients should be given clear advice about adjusting medication doses in the event of a severe persistent hypertensive response. It has previously been suggested that medication adjustments in known hypertensive patients at altitude should be considered in accordance with sea-level practices for patients with hypertensive urgency or emergency at altitude [2], namely:

1. If SBP is greater than 180 mmHg or BDP is greater than 120 mmHg and there is evidence of side effects from these elevated pressures, including vision changes, shortness of breath, chest pain, or altered mental status.
2. SBP is greater than 220 mmHg or DBP is greater than 140 mmHg in the absence of symptoms.

Given the possibility of end-organ damage that our case illustrates, we suggest that descent should also be considered in these circumstances if there is not an immediate response to increasing antihypertensive medications. Furthermore, in patients who are not known to have hypertension, we would suggest that immediate descent should be recommended in the two scenarios outlined above. Commencing new anti-hypertensives in a remote environment, with no knowledge of underlying renal function nor having the ability to predict or monitor possible renal injury, cannot be recommended.

Ultimately, given the increasing popularity of high-altitude travel and the relatively high incidence of hypertension, further research is warranted to evaluate this problem. Studies should focus on identifying risk factors for large increases in blood pressure, and in particular, whether a hypoxic stimulus at sea level predicts the altitude hypertensive response. In addition the persistence of altitude-induced hypertension on return to normoxia should be evaluated, as should implications for future hypertensive risk at sea level. Finally, clinical trials to determine the most appropriate treatment for clinically significant blood pressure elevations in this environment are needed.

## Conclusion

This case report is the first documented case of a hypertensive crisis at altitude as well as the first report of the occurrence of AKI in the context of altitude-related hypertension. This report challenges the view that transient rises in blood pressure at altitude are without immediate risk. Further studies are needed to identify the prevalence of severe hypertension at altitude, to identify risk factors and to clarify the most appropriate treatment. In the absence of sufficient evidence, we recommend a conservative approach of screening at risk patients, increasing medication in known hypertensives with severe hypertension, and consideration of descent in previously normotensive subjects who develop severe and sustained hypertension.

## Abbreviations

AMS: acute mountain sickness
BP: blood pressure
EBC: Everest Base Camp
ECG: electrocardiogram
ECHO: echocardiogram
DBP: diastolic blood pressure
MRI: magnetic resonance imaging
SBP: systolic blood pressure
